# Microencapsulation of *Elsholtzia ciliata* Herb Ethanolic Extract by Spray-Drying: Impact of Resistant-Maltodextrin Complemented with Sodium Caseinate, Skim Milk, and Beta-Cyclodextrin on the Quality of Spray-Dried Powders

**DOI:** 10.3390/molecules24081461

**Published:** 2019-04-13

**Authors:** Lauryna Pudziuvelyte, Mindaugas Marksa, Valdas Jakstas, Liudas Ivanauskas, Dalia M. Kopustinskiene, Jurga Bernatoniene

**Affiliations:** 1 Institute of Pharmaceutical Technologies, Medical Academy, Lithuanian University of Health Sciences, Sukileliu pr. 13, Kaunas LT-50161, Lithuania; lauryna.pudziuvelyte@lsmuni.lt (L.P.); valdas.jakstas@lsmuni.lt (V.J.); daliamarija.kopustinskiene@lsmuni.lt (D.M.K.); 2 Department of Drug Technology and Social Pharmacy, Medical Academy, Lithuanian University of Health Sciences, Sukileliu pr. 13, Kaunas LT-50161, Lithuania; 3 Department of Analytical and Toxicological Chemistry, Medical Academy, Lithuanian University of Health Sciences, Sukileliu pr. 13, Kaunas LT-50161, Lithuania; minzedas@gmail.com (M.M.); liudas.ivanauskas@lsmuni.lt (L.I.); 4 Department of Pharmacognosy Medical Academy, Lithuanian University of Health Sciences, Sukileliu pr. 13, Kaunas LT-50161, Lithuania

**Keywords:** *Elsholtzia ciliata*, extract, essential oil, spray-drying, microcapsules

## Abstract

Spray-drying is the most popular encapsulation method used for the stabilization and protection of biologically active compounds from various environmental conditions, such as oxidation, moisture, pH, and temperature. Spray-drying increases the bioavailability of the natural active compounds and improves the solubility of low-soluble compounds. The aim of this work was to study the effects of different wall materials and optimize wall material solution’s composition on physicochemical properties of microcapsules loaded with phenolics, extract rich in volatile compounds and essential oil from *Elsholtzia ciliata* herb. For encapsulation of elsholtzia and dehydroelsholtzia ketones, more suitable wall materials were used—beta-cyclodextrin and sodium caseinate. Four phenolics—sodium caseinate, skim milk, beta-cyclodextrin, and resistant-maltodextrin—were used. A D-optimal mixture composition design was used to evaluate the effect of wall material solution’s composition using sodium caseinate (0.5–1 g), skim milk (6–10 g), resistant-maltodextrin (8–12 g), and beta-cyclodextrin (0.5–1 g) for the encapsulation efficiency, drying yield, and physicochemical properties. The optimal mixture composition was 0.54 g of sodium caseinate, 10 g of skim milk, 8.96 g of resistant-maltodextrin, and 0.5 g of beta-cyclodextrin. These encapsulating agents had a good performance in the microencapsulation of *E. ciliata* ethanolic extracts by the spray-drying technique. It is proven that the produced microparticles have a good potential to be included in various pharmaceutical forms or food supplements.

## 1. Introduction

Extracts with various chemical compounds are produced from different medicinal plants. These extracts frequently show antioxidant, anti-inflammatory, antimicrobial, and antiviral effects. *Elsholtzia ciliata* (Vietnamese Balm) constitutes attractive raw materials for pharmaceutical and food industries due to its volatile compounds [1], polyphenol contents [2], and anticancer activity [3]. The main interest in *E. ciliata* is due to its popularity as an ingredient in traditional cuisine and for its medical purposes [1,4]. The consumption of polyphenols and essential oils in extracts is well known for being beneficial for human health. As in vitro, animal, human, and epidemiologic studies have shown, it has correlated with a lower risk of cardiovascular diseases, neurodegenerative disorders, obesity, osteoporosis, gastrointestinal problems, and cancer, mainly because of their antioxidant properties [5,6,7,8,9,10].

According to the structural aspect, polyphenols fall into many different families, including flavonoids anthocyanins, coumarins, lignins, tannins, acids, and phenols [5,11,12,13]. This structural diversity results in a large variability of the physicochemical properties influencing the extraction of polyphenols [13]. Solubility is considered to be the key aspect when the effects of polyphenols and essential oils in vivo are assessed. Poor solubility influences the bioavailability of polyphenols and essential oils. Only 1% to 10% of total polyphenol intake is detected in urine and plasma samples [5]. A method for increasing bioavailability and reducing inter- and intra-individual variability should be analyzed due to poor solubility of polyphenols and essentials oils, and the difficulties of oral administration. Also, polyphenols and essential oils are sensitive to various environmental conditions such as temperature, pH, and oxidation, which may reduce their bioavailability, content, nutritional value, and storage period [8,9].

In recent years, microencapsulation of food, pharmaceutical, personal care, and cosmetic product ingredients has become very attractive, popular, and interesting, and production processes that are associated with it have become technologically feasible [14]. According to Dias et al. [15], the most popular encapsulation technique for bioactive components and probiotics in food has been spray-drying (over 110 publications over the past 2 years (2015–2016)) [9,16]. Spray-drying is a low-cost, fast, effective, and available microencapsulation technique used for the preparation of powders due to its easy industrialization. Furthermore, it allows a continuous production [17,18,19].

**Figure 1:**
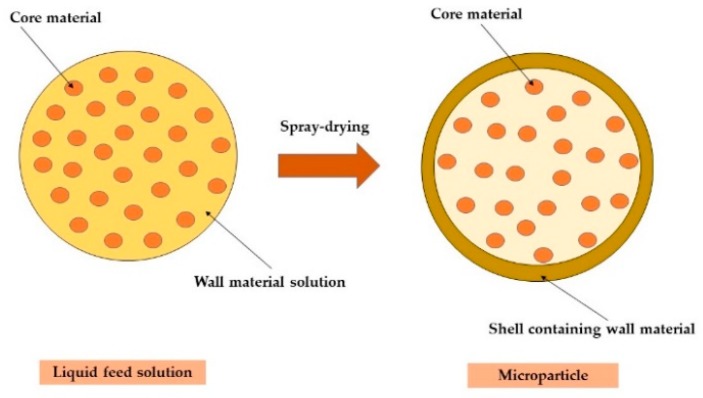
Scheme showing the shell forming around particles during spray-drying.

Spray-drying microencapsulation technology is beneficial for the protection of active compounds that are sensitive to free radical degradation, light, and oxygen [20,21,22,23,24,25]. Spray-dried powders have low water activity, excellent reconstitutional characteristics, and are suitable for transportation and storage [26]. The main protective role of microencapsulation technology is to form a membrane/shell (wall material) around particles or droplets of active ingredients that are being encapsulated (core material) (Figure 1) [14,20].

Depending on the polymer used, microencapsulation may control organoleptic modifications and increase the solubility/dissolution rate of the spray-dried product [8]. A good encapsulating agent (wall material) should have emulsifying and film forming properties, display low hygroscopicity, have low viscosity at high solid contents, resistance to the gastrointestinal tract, be biodegradable, non-toxic, low-cost, bland in flavor/tasteless, soluble in aqueous solvents, and food-grade [19]. However, no single encapsulant possesses all these properties, and because of that, two or more encapsulants must often be used in particular combinations [19]. The type of wall material affects the quantification of active substances due to the interaction between active compounds and wall material [9]. The main groups of wall materials for microencapsulation are lipids (glycerides, phospholipids, waxes, etc.), proteins (gluten, whey proteins, isolates, etc.), and carbohydrates (starch, cellulose and their derivatives, plant exudates, etc.) [23,27]. Maltodextrin and gum arabic are used as encapsulants in spray-drying technique more commonly than other materials [19]. Maltodextrin is a hydrolyzed starch which is suitable for encapsulation of active ingredients because it has relatively low viscosity at high concentrations, a good protection against oxidation, and low cost [8,9]. Gum arabic is a suitable encapsulant for spray-drying because of its high solubility, good emulsifying properties, and low viscosity [9,28,29]. There are only a few methods that improve the solubility of phenolic compounds and essential oils, which in turn upgrade the said bioactive compound’s bioavailability when taken orally [9,30,31]. Skim milk and sodium caseinate are suitable encapsulants for microencapsulation because of their low viscosity, high solubility, and good emulsifying properties. According to Ratchathani et al. [32], skim milk powder as an encapsulating agent keeps the moisture content of fish oil microcapsule, and this feature is obtained by spray-drying in the range of 2.33–4.84%. A relatively new water-soluble fiber, resistant-maltodextrin, can be used as an encapsulating agent for spray-drying. Resistant-maltodextrin is a randomly linked alpha-glucoside oligosaccharide and it has a low glycemic index (10% of that of maltodextrin) [16]. Most of the research work on resistant-maltodextrin has focused on its nutritional benefits. However, we have only come across a few recent reports on the use of resistant-maltodextrin as an encapsulant using spray- and freeze-drying processes, which mainly focus on stability, sensory profiles [33], and bioflavonoid naringin encapsulation [16].

To the best of our knowledge, there is no available information on an eligible wall material for the encapsulation of *E. ciliata* essential oil and ethanolic extract. Since the coating behavior of each wall material is different, the suitability of a wall material for encapsulation needs to be evaluated due to the lack of knowledge about the compatibility of the encapsulate and its coating properties. This study will provide knowledge on selecting wall materials in order to produce a powder containing bioactive compounds and other related research. The influence of wall material composition for microencapsulation was evaluated. Sodium caseinate, skim milk, maltodextrin, resistant-maltodextrin, gum arabic, and beta-cyclodextrin were chosen as the encapsulating agents (wall materials). Powder quality tests and encapsulation efficiency of the main active compounds contained in *E. ciliata* ethanolic extract and essential oil (core materials) were the main parameters for the evaluation.

## 2. Results and Discussion

### 2.1. Influence of Wall Material Components on the Physicochemical Properties

Core material (ethanolic extract and essential oil) for spray-drying was prepared as described in section materials and methods, using extraction methods that were developed before to achieve the highest quantities of rosmarinic acid (RA), chlorogenic acid (CA), apigenin (AP), elsholtzia ketone (EK), dehydroelsholtzia ketone (DK), and total phenolics content (TPC) [34].

**Figure 2:**
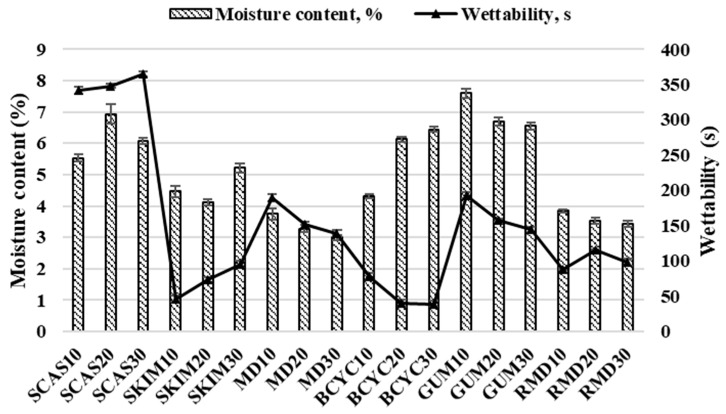
Moisture content (%) and wettability (s) values obtained in spray-dried powders with different wall materials. SCAS (sodium caseinate), SKIM (skim milk), MD (maltodextrin), BCYC (beta-cyclodextrin), GUM (gum arabic), RMD (resistant-maltodextrin). Numbers 10, 20, and 30 mean concentration (%) of wall materials in liquid feed solution composition for spray-drying.

At the first stage of the experiment, the selection of the most suitable encapsulant agent for core material microencapsulation was carried out. Sodium caseinate, skim milk, resistant-maltodextrin, maltodextrin, gum Arabic, and beta-cyclodextrin were selected as potential wall materials. The concentration of encapsulating agents in liquid feed solution for spray-drying varied from 10% to 30%. In all experiments inlet temperature was 160 °C, the outlet temperature was 80–90 °C, and spray flow feed rate was 30 mL/min. The data in Figure 2 shows the impact of the amount of encapsulating agent in a liquid feed solution for spray-drying on the moisture content and wettability.

Moisture content is a relevant parameter in the determination of quality and shelf life of powder [35,36]. A high moisture content of powder can cause a stickiness in the particles. The moisture content of spray-dried powders ranged from 2.99% ± 0.07% to 7.6% ± 0.14%, and was the highest when wall material contained gum arabic (10%) and the lowest for maltodextrin (30%). The moisture content decreases with increasing the amount of wall material in liquid feed solution for spray-drying: maltodextrin 10–3.78% ± 0.18%, 20–3.28% ± 0.11%, 30–2.99% ± 0.07%; gum arabic 10–7.6% ± 0.14%, 20–6.7% ± 0.14%, 30–6.55% ± 0.11%; and resistant-maltodextrin 10–3.85% ± 0.05%, 20–3.54% ± 0.1%, 30–3.43% ± 0.09%. Our results are in line with the data of Ferrari et al. [37] on the spray-dried blackberry powders when using maltodextrin as wall material. In this research, when a higher concentration of maltodextrin was used, a powder with a lower moisture content was obtained. According to our study, using beta-cyclodextrin as wall material, the moisture content in spray-dried powder increases: 10–4.31% ± 0.06%, 20–6.13% ± 0.08% and 30–6.45% ± 0.09%.

The ability of the powder particles to overcome the surface tension between themselves and water or time required for the powder to become completely wet is called wettability [38]. The time required for the powders to become completely moist varied from 40 ± 4.95 to 348 ± 2.83 s. Shorter time was reached when beta-cyclodextrin 20% was used as a wall material and it was prolonged when sodium caseinate 20% was used. The wettability of the powder increased with increasing concentration of wall material. Higher concentrations (10% > 20% > 30%) of wall materials increased wettability (maltodextrin 189 > 151 > 139 s, beta-cyclodextrin 78 > 40 > 39 s, gum arabic 193 > 157 > 144 s, respectively). Opposite results were obtained by A-sun et al. [38], that higher maltodextrin concentrations decrease wettability. Increasing skim milk concentrations (10% > 20% > 30%) led to a significant decrease in wettability (46, 73, and 95 s, respectively). This can be explained by the fact that skim milk acts as a bulking agent that affects porous structure by making less porous powders.

**Figure 3:**
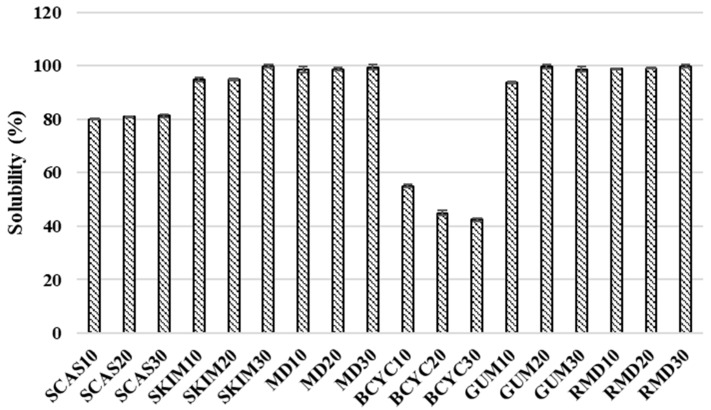
Solubility (%) obtained in spray-dried powders with different wall materials. SCAS (sodium caseinate), SKIM (skim milk), MD (maltodextrin), BCYC (beta-cyclodextrin), GUM (gum arabic), RMD (resistant-maltodextrin). Numbers 10, 20, and 30 mean concentration (%) of wall materials in liquid feed solution composition for spray-drying.

Spray-dried powder solubility ranged from 42.5 ± 0.49 to 99.9 ± 0.65% and was the highest when wall material contained resistant-maltodextrin (20%) and the lowest for beta-cyclodextrin (30%) (Figure 3).

Maltodextrin is widely used as an encapsulant in spray-drying because it has high values of solubility in water and high glass transition temperature [39]. Higher concentrations (10% > 20% > 30%) of wall material influenced higher solubility of spray-dried powders (sodium caseinate 80.05% > 80.9% > 81.25%, skim milk 95% > 95% > 99.9%, respectively), however using beta-cyclodextrin in the same concentration range solubility decreases (55%, 45%, and 42.5%, respectively).

Quality control parameters for spray-dried powders such as Carr index and Hausner ratio should be considered. Lower Carr index shows a better powder flowability, whereas high Hausner ratio reflects the spray-dried powder’s cohesiveness and it being less capable of flowing freely.

**Figure 4:**
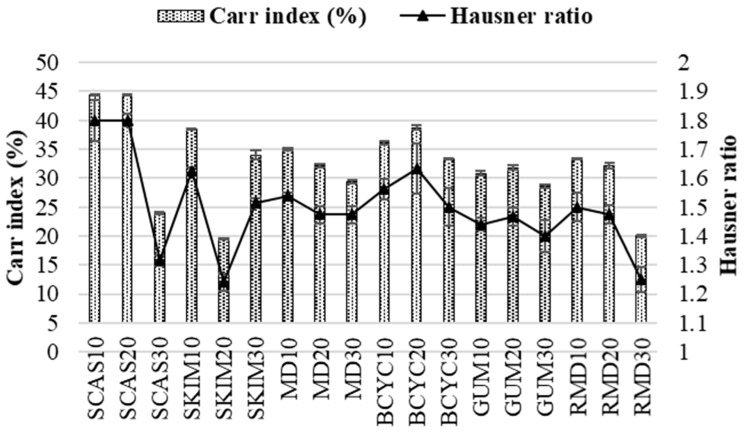
Carr index (%) and Hausner ratio values obtained in spray-dried powders with different wall materials. SCAS (sodium caseinate), SKIM (skim milk), MD (maltodextrin), BCYC (beta-cyclodextrin), GUM (gum arabic), RMD (resistant-maltodextrin). Numbers 10, 20, and 30 mean concentration (%) of wall materials in liquid feed solution composition for spray-drying.

Results determined in our study are presented in Figure 4. The Carr index varied from 19.44% ± 0.13% to 44.44% ± 0.08% and Hausner ratio from 1.241 ± 0.03 to 1.8 ± 0.07. The highest Carr index and Hausner ratio was when wall material contained sodium caseinate (10% and 20%) and the lowest for skim milk (20%).

### 2.2. Influence of Wall Material Components on the Encapsulation Efficiency of Active Compounds

**Figure 5:**
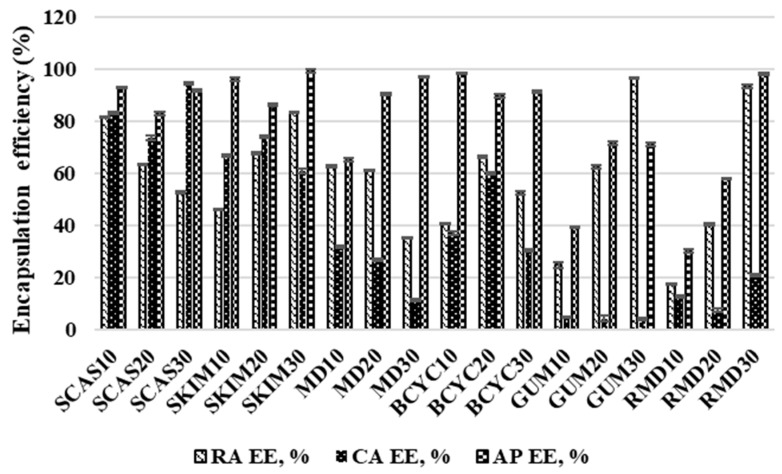
Encapsulation efficiency (%) of RA (rosmarinic acid), CA (chlorogenic acid) and AP (apigenin) obtained in spray-dried powders with different wall materials. SCAS (sodium caseinate), SKIM (skim milk), MD (maltodextrin), BCYC (beta-cyclodextrin), GUM (gum arabic), RMD (resistant-maltodextrin). Numbers 10, 20, and 30 mean concentration (%) of wall materials in liquid feed solution composition for spray-drying.

**Figure 6:**
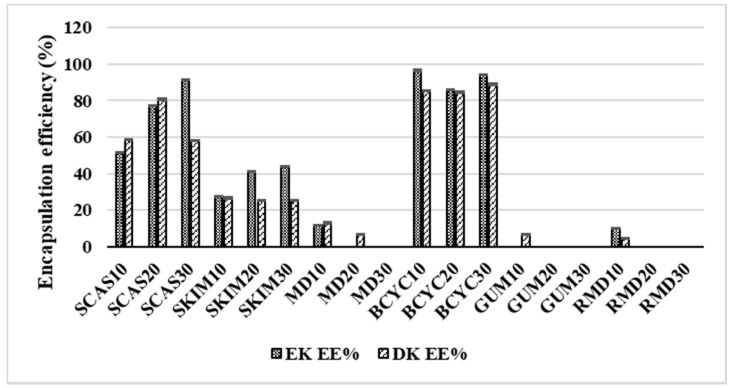
Encapsulation efficiency (%) of EK (elsholtzia ketone) and DK (dehydroelsholtzia ketone) obtained in spray-dried powders with different wall materials. SCAS (sodium caseinate), SKIM (skim milk), MD (maltodextrin), BCYC (beta-cyclodextrin), GUM (gum arabic), RMD (resistant-maltodextrin). Numbers 10, 20, and 30 mean concentration (%) of wall materials in liquid feed solution composition for spray-drying.

**Figure 7:**
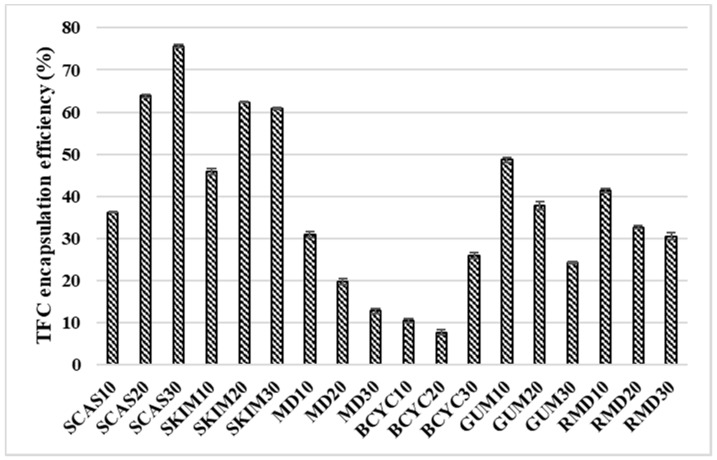
Encapsulation efficiency (%) of TFC (total phenolics content) obtained in spray-dried powders with different wall materials. SCAS (sodium caseinate), SKIM (skim milk), MD (maltodextrin), BCYC (beta-cyclodextrin), GUM (gum arabic), RMD (resistant-maltodextrin). Numbers 10, 20, and 30 mean concentration (%) of wall materials in liquid feed solution composition for spray-drying.

The data presented in Figure 5, Figure 6, and Figure 7 show the impact of the concentration of wall material liquid feed solution on the EE of the main active compounds in ethanolic extract and essential oil. The RA EE of spray-dried powders varied from 17.69% ± 0.09% to 93.33% ± 0.62% and was the highest when wall material contained 30% resistant-maltodextrin and the lowest when it contained 10% resistant-maltodextrin (Figure 4).

The EE of RA increases with increasing concentration (10% > 20% > 30%) of wall material in liquid feed solution for spray-drying: skim milk 45.98%, 67.71%, 83.33%, gum arabic 24.89%, 62.47%, 96.36%, resistant-maltodextrin 17.69%, 40.56%, and 93.33%, respectively). However, the EE of RA decreases using sodium caseinate (81.33% > 63.48% > 52.71%) and maltodextrin (62.75% > 61.25% > 35.15%) as coating materials at the same range of concentrations (10%, 20%, and 30%). The CA EE of spray-dried powders varied from 4.07% ± 0.52% to 94.46% ± 0.4% and was the highest when wall material contained sodium caseinate (30%) and the lowest when gum arabic (30%) was used (Figure 5). The EE of CA decreases with increasing concentration (10% > 20% > 30%) of wall material in liquid feed solution for spray-drying: maltodextrin 32.02%, 26.81%, 11.32%, gum arabic 4.93%, 4.19%, and 4.07%, respectively. The AP EE of spray-dried powders varied from 30.13 ± 0.69 to 99.31 ± 0.64% and was the highest when wall material contained skim milk (30%) and the lowest when resistant-maltodextrin (10%) was used (Figure 5). The EE of AP increases with increasing concentration (10% > 20% > 30%) of wall material in liquid feed solution for spray-drying: maltodextrin 65.08%, 90.38%, 97.16%, resistant-maltodextrin 12.66%, 7.51%, and 98.2%, respectively. The EK EE of spray-dried powders varied from 10.29% ± 0.2% to 96.59% ± 0.4% and was the highest when wall material contained beta-cyclodextrin (10%) and the lowest when it contained resistant-maltodextrin (10%) (Figure 6). Using sodium caseinate and skim milk in the concentration range of 10%, 20%, and 30%, EK EE increases accordingly—51.47, 77.27, 91.37 and 27.64, 41.17, 43.96. Gum arabic in all concentrations did not encapsulate EK.

The DK EE of spray-dried powders ranged from 4.4% ± 0.24% to 88.9% ± 0.42% and was the highest when wall material contained beta-cyclodextrin (30%) and the lowest when resistant-maltodextrin (10%) was used (Figure 6). The EE of DK decreases with increasing concentration (10% > 20% > 30%) of wall material in liquid feed solution for spray-drying: skim milk 26.64%, 25.51%, 25.27%, maltodextrin 12.91%, 7.13%, (10% of wall material didn’t encapsulate DK), respectively.

Figure 7 shows that the TPC EE% varied from 7.74% ± 0.69% to 75.59% ± 0.36% and it was the highest when the wall material contained 30% of sodium caseinate (SCAS30) and the lowest when the wall material contained 20% of beta-cyclodextrin (BCYC20). The TFC EE (%) increases when the wall material concentrations are in the range of 10% > 20% > 30% (using sodium caseinate (SCAS) and skim milk (SKIM)). The TFC EE (%) decreases when the concentration of wall material increases (for example, using maltodextrin (MD), gum arabic (GUM), and resistant-maltodextrin (RMD)) (Figure 7).

According to our results for the next stage of experiments, sodium caseinate, skim milk, beta-cyclodextrin, and resistant-maltodextrin were chosen. Sodium caseinate and skim milk are suitable for rosmarinic acid, chlorogenic acid, and apigenin microencapsulation because the EE of these active compounds was more than 50%. For EK and DK microencapsulation only sodium caseinate, skim milk, beta-cyclodextrin were suitable. Gum arabic and maltodextrin show low EE of these ketones.

### 2.3. The Influence of Wall Material Containing Four Encapsulants on the Physicochemical Properties and Encapsulation Efficiency of Active Compounds

At the second experiment stage, the selection of an optimal ratio of wall materials in the liquid feed solution was carried out. Parameters for D-optimal mixture design in preparing liquid feed solution for spray-drying were prepared by using Design-Expert Version 9.0 software as shown in Table 1. The regression coefficients, final equations are presented in Table 2. Liquid feed solution contains four encapsulants: sodium caseinate, skim milk, resistant-maltodextrin, and beta-cyclodextrin. These encapsulants were chosen to be the variables toward the responses: EE of RA, CA, AP, EK, and DK. The total amount of wall material (a mixture of four encapsulants) was 20 g, the inlet temperature was 160 °C, the outlet temperature was 80–90 °C, and the spray flow feed rate was 30 mL/min.

The moisture content of spray-dried powders ranged from 3.71% ± 0.05% to 5.92% ± 0.08%. The moisture of spray-dried powders using four materials as encapsulating agents for spray-drying solution decreased by 1.2 times compared to when only gum arabic was used (7.6% ± 0.14%) (Figure 2, Table 3). The time required for the spray-dried powders to become completely wet ranged from 62 ± 0.35 to 105 ± 0.32 s and it was three times shorter when four materials for the spray-drying were used, compared to only using sodium caseinate (348 ± 2.83 s) as encapsulating agent (Figure 2, Table 3). Using solution which contains four materials for spray-drying, the solubility varied from 82.5% ± 0.03% to 99.8% ± 0.01% and it was approximately 2 times higher than only using beta-cyclodextrin (42.5% ± 0.49%) as encapsulating agent (Figure 3, Table 3). According to the results, better values of Carr index (from 24.44% ± 0.07% to 37.14% ± 0.03%) and Hausner ratio (from 1.324 ± 0.07 to 1.591 ± 0.03) were obtained by using four encapsulants for spray-drying rather than using, e.g., only sodium caseinate (44.44% ± 0.08% and 1.8 ± 0.07, respectively) (Figure 4, Table 3). Spray-dried powders characteristics were improved using wall material mixture optimization. Optimization was suitable to improve solubility and wettability of powders (get lower values tan using only one encapsulant for spray-drying).

The yield of spray-dried powders varied from 44.19% ± 0.33% to 66.97% ± 0.98% and was the highest when the composition was 0.5 g of sodium caseinate, 7 g of skim milk, 12 g of resistant-maltodextrin, and 0.5 g of beta-cyclodextrin. The lowest yield was obtained when composition of wall materials was 1 g of sodium caseinate, 8.13 g of skim milk, 10.13 g of resistant-maltodextrin, and 0.75 g of beta-cyclodextrin.

**Figure 8:**
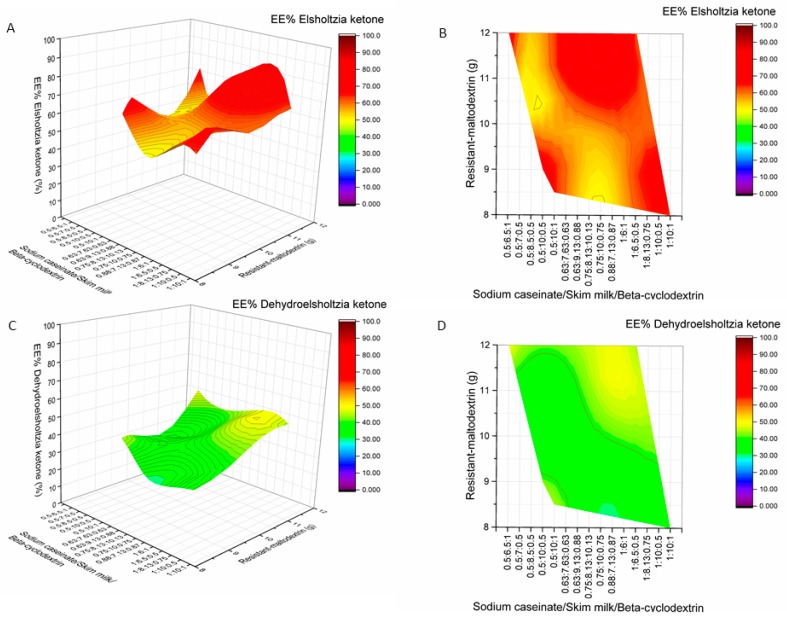
Influence of wall material component (resistant-maltodextrin supplemented with skim milk, sodium caseinate, and beta-cyclodextrin) amounts on encapsulation efficiencies (EE%) of elsholtzia ketone (**A**,**B**) and dehydroelsholtzia ketone (**C**,**D**). A, C—surface plots; B, D—contour plots.

**Figure 9:**
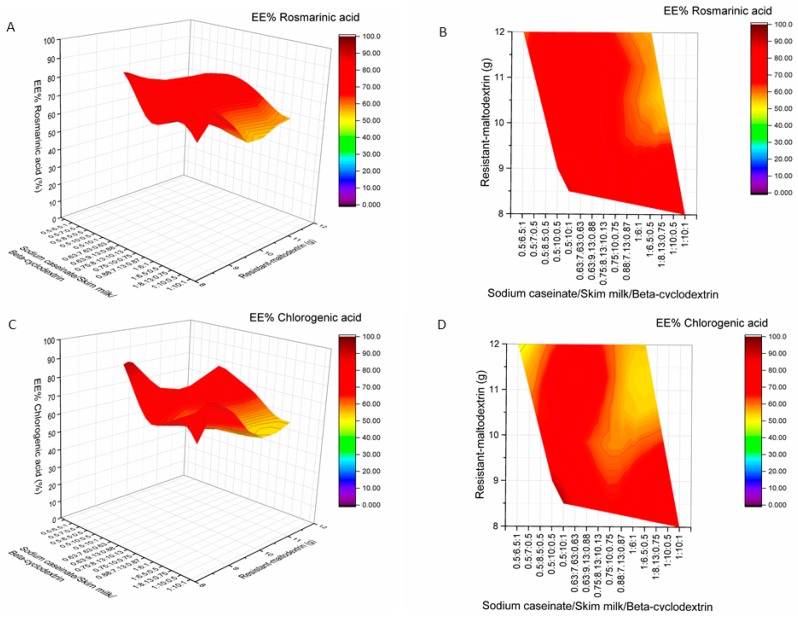
Influence of wall material component (resistant-maltodextrin supplemented with skim milk, sodium caseinate, and beta-cyclodextrin) amounts on encapsulation efficiencies (EE%) of rosmarinic acid (**A**,**B**) and chlorogenic acid (**C**,**D**). A, C—surface plots; B, D—contour plots.

**Figure 10:**
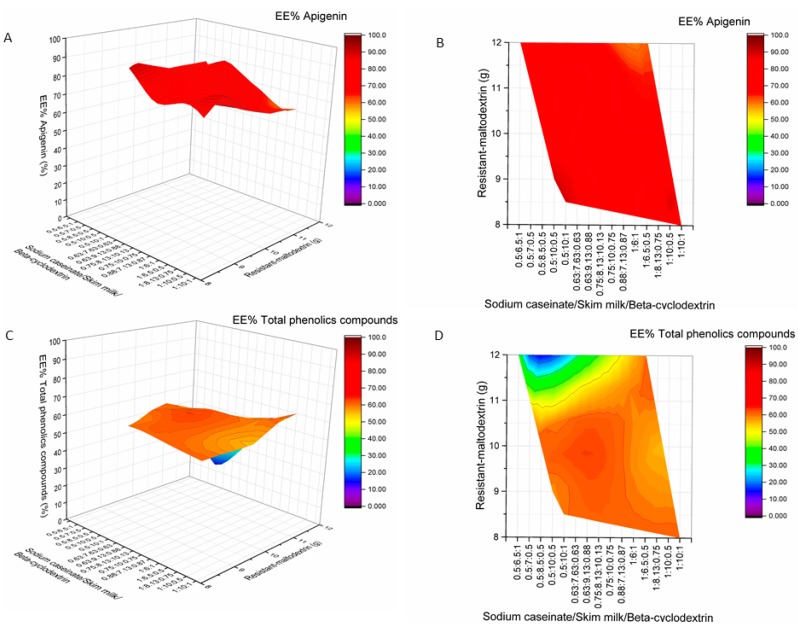
Influence of wall material component (resistant-maltodextrin supplemented with skim milk, sodium caseinate, and beta-cyclodextrin) amounts on encapsulation efficiencies (EE%) of apigenin (**A**,**B**) and total phenolics compounds (**C**,**D**). A, C—surface plots; B, D—contour plots.

The highest EE of RA (89.96%), CA (93.24%), AP (89.65%), EK (64.94%), DK (45.74%), and TPC (60.12%) were achieved when 0.5 g of sodium caseinate, 10 g of skim milk, 8.5 g of resistant-maltodextrin, and 1 g of beta-cyclodextrin were used as encapsulants for spray-drying (Figure 8, Figure 9 and Figure 10). The product yield of in these conditions was 56.04%. The lowest EE of RA was 46.37% (02M, Table 5, Figure 9), CA—47.87% (19M, Table 5, Figure 9), AP—55.14% (5M, Table 5, Figure 10), EK—44.38% (8M, Table 5, Figure 8), and DK—28.13% (M9, Table 5, Figure 8). In all samples 02M, 05M, and 19M included the same amount of wall materials: 12 g of resistant-maltodextrin and 1 g of beta-cyclodextrin. Other wall material amounts varied from 0.5 g to 1 g of sodium caseinate and from 6 g to 6.5 g of skim milk. According to the results, higher amounts than 8 g of resistant-maltodextrin could impact the lower EE (%) of phenolic compounds, EK, and DK. Also, smaller amounts than 10 g of skim milk in the mixture of wall materials could result in lower EE (%) of active compounds.

The numerical optimization of wall material amounts using the desirability function has been performed. The optimization parameters are presented in Table 4.

The obtained results showed that the optimal composition of *E. ciliata* microcapsules has been determined as follows: 0.54 g of sodium caseinate, 10 g of skim milk, 8.96 g of resistant-maltodextrin, and 0.5 g of beta-cyclodextrin. All experimental values did not significantly differ from the predicted values (*p* > 0.05). According to the results, this composition of wall materials is more suitable for encapsulation of phenolics. EE (%) of RA, CA, and AP was more than 85%, which is higher than the EE of EK and DK, 67.38% and 35.12%, respectively. In order to obtain the highest EE values of ketones it is important to choose suitable conditions for spray-drying, such as temperature and feed flow. In the next stage of the study, it is necessary to optimize conditions of spray-drying to increase EE of ketones.

### 2.4. Scanning Electron Microscopy of Spray-Dried Powders

**Figure 11:**
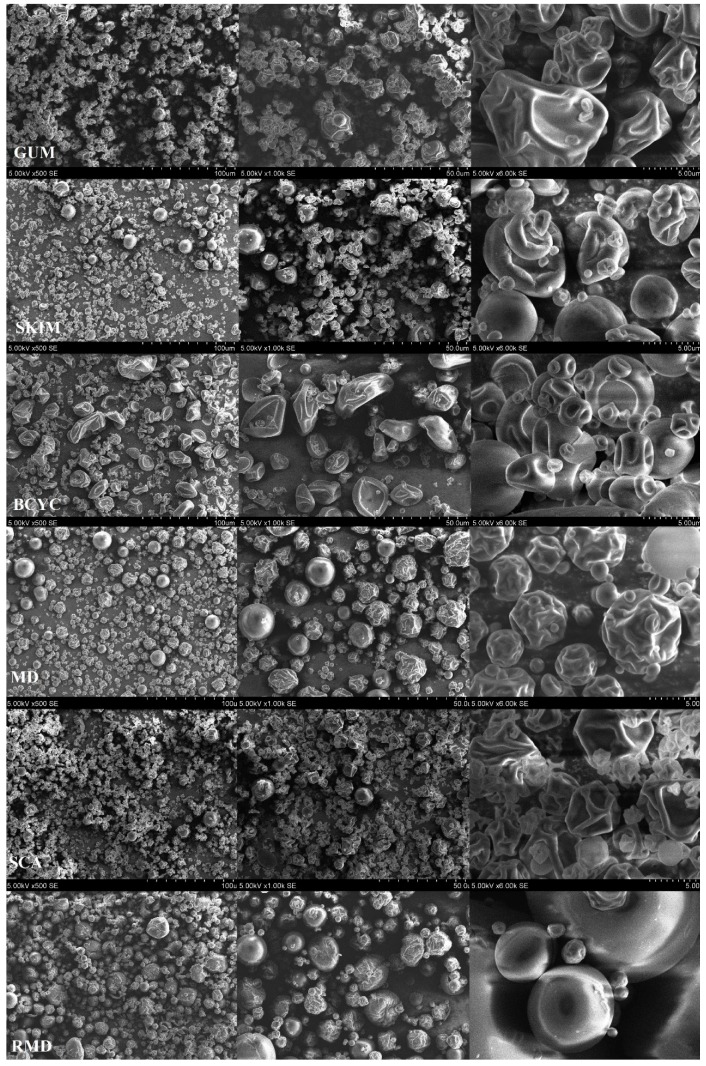
Scanning electron microscopy (SEM) images of microcapsules using 500×, 1000×, and 6000× magnifications: GUM (gum arabic), SKIM (skim milk), BCYC (beta-cyclodextrin), MD (maltodextrin), SCA (sodium caseinate), RMD (resistant-maltodextrin). Images were prepared using microparticles which were spray-dried using 20% of wall materials.

The *E. ciliata* ethanolic extract and essential oil microcapsules using GUM, SKIM, BCYC, MD, SCA, and RMD as encapsulating agents were obtained by spray-drying. The microcapsules’ morphologies were observed by using scanning electron microscopy (SEM) (Figure 11). Analyzing the micrographs, the most common morphology of microcapsules was semi-spherical. There are two types of microparticle morphologies: particles with smooth surface and particles with a concave and folded surface.

**Figure 12:**
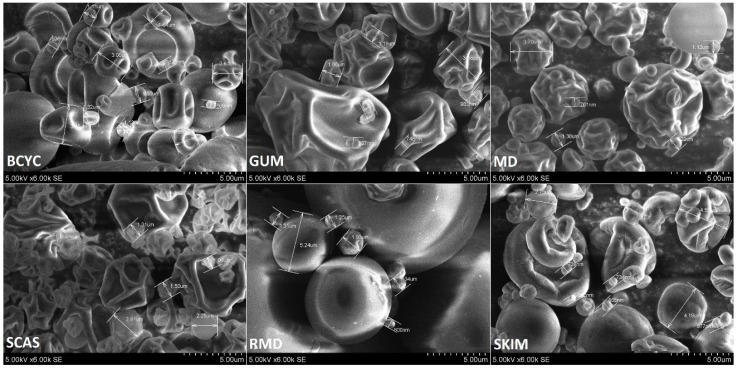
SEM images of microcapsules using 6000× magnifications with measured diameters: GUM (gum arabic), SKIM (skim milk), BCYC (beta-cyclodextrin), MD (maltodextrin), SCAS (sodium caseinate), RMD (resistant-maltodextrin). Images were prepared using microparticles which were spray-dried using 20% of wall materials.

GUM and BETA microparticles showed more folded and dented particles. Diameters of GUM microparticles varied from about 748 nm to 3.88 µm, and BETA from 599 nm to 345 µm (Figure 12).

SKIM microparticles were semi-spherical and there were particles that looked compressed. Diameters of these particles varied from about 562 nm to 4.59 µm and more. MD microparticles showed more semi-spherical particles with smooth and wrinkled surfaces in diameters range from about 761 nm to 370 µm and more. In Diameters of SCA microparticles varied from about 1.06 nm to 2.81 µm and most of the particles were deeply wrinkled (Figure 11 and Figure 12). In addition, the results on Figure 11 show that RMD particles presented a different morphology when compared to particles spray-dried with other wall materials. Diameters of RMD microparticles varied from about 800 nm to 5.62 µm and more (Figure 12). The majority of RMD microparticles had a semi-spherical appearance with smooth and less wrinkled surfaces which was important to confer a better encapsulation of the *E. ciliata* extract. This may be due to the excellent film forming property by the RMD. The water evaporation rates observed in the spray-drying process is most likely the reason of morphological irregularities that have appeared on the surface of the microparticles. Lower inlet temperature of spray-drying process resulted in irregular microparticle form with shrunk, concave surfaces, while higher inlet temperature resulted in smoother, tighter microparticles [20,40].

**Figure 13:**
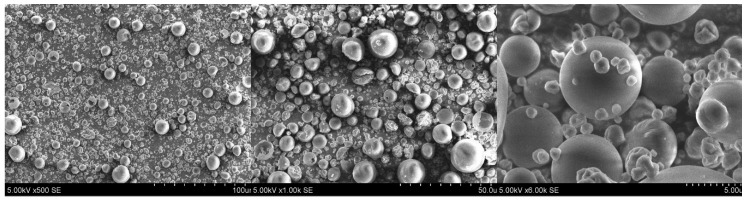
SEM images of microcapsules, which were spray-dried using skim milk, sodium caseinate, beta-cyclodextrin, and resistant-maltodextrin as wall materials (magnification 500×, 1000×, and 6000×).

In Figure 13 fragmented particles of skim milk, sodium caseinate, beta-cyclodextrin, and resistant-maltodextrin are shown. The majority of spray-dried microparticles were spherical in shape, almost equal in size, and had a smooth surface without or with a few wrinkles on the surface. Diameters of microparticles varied from about 404 nm to 5.29 µm and more. Using four encapsulating agents improves smaller particles’ size and forms smooth surfaces. Using skim milk, sodium caseinate, beta-cyclodextrin, and resistant-maltodextrin in one encapsulating agent solution produced a smoother surface and spherical shape particles compared to when only one encapsulating agent for ethanolic extract encapsulation by spray-drying was used.

## 3. Materials and Methods

### 3.1. Materials

Dried *Elsholtzia ciliata* herb was purchased from “Zolynu namai” (Vilnius, Lithuania). A voucher specimen (L 17710) has been deposited at the Herbarium of the Department of Drug Technology and Social Pharmacy, Lithuanian University of Health Sciences.

HPLC eluents: formic acid, phosphoric acid, and acetonitrile were purchased from Sigma-Aldrich, (Steinheim, Germany), methanol from Carl Roth GmbH, (Karlsruhe, Germany). Standards for HPLC analysis: chlorogenic acid, rosmarinic acid, apigenin, and eugenol were purchased from Extrasynthese, (Genay, France). Wall material compounds: sodium caseinate, skim milk, maltodextrin, gum arabic, beta-cyclodextrin were purchased from Sigma-Aldrich, (Steinheim, Germany), resistant-maltodextrin (Promitor 85 ™) was purchased from Bang & Bonsomer, (Vilnius, Lithuania). Folin-Ciocalteu reagent, gallic acid monohydrate, and sodium carbonate were purchased from Sigma-Aldrich GmbH (Buchs, Switzerland). Extraction solvent ethanol (96%) was purchased from Vilniaus degtine, (Vilnius, Lithuania). Water used in HPLC and for the sample preparation was produced with a Millipore Super Purity Water System, (Sigma-Aldrich Corp., St. Louis, MO, USA).

### 3.2. Preparation of E. ciliata Ethanolic Extract

Before the extract preparation, *E. ciliata* herbal material was grounded in a laboratory mill. Powdered material was then extracted with 70% (*v*/*v*) ethanol in a conical flask by ultrasound-assisted extraction performed in an ultrasound bath (Bandelin electronic GmbH & Co.KG, Berlin, Germany) at 25 °C for 30 min. These conditions were established as the best for the extraction of the main compounds from *E. ciliata* herb in our previous study [34]. The samples were centrifuged for 10 min at 4200× *g*, followed by decantation of the supernatant. The prepared extract was filtered using a vacuum filter and stored in refrigerator at +4 °C until further use.

### 3.3. Preparation of E. ciliata Essential Oil

The essential oil from dried *E. ciliata* herb was prepared using the same hydrodistillation conditions obtained in our previous study [34]. A dried grounded herb (30 g) was blended with 500 mL purified water and submitted to extraction for four hours until no more essential oil was obtained. The light yellow-colored essential oils with strong specific aroma were collected with water and stored in refrigerator at +4 °C until further use.

### 3.4. Liquid Feed Preparation for Spray-Drying

The solution for spray-drying consisted of the solution of wall material—resist-maltodextrin, maltodextrin, skim milk, beta-cyclodextrin, gum arabic, sodium caseinate and active substances—and ethanolic extract and essential oil prepared from *E. ciliata* dried herb. The wall material solutions were prepared by wetting the required amounts of resistant-maltodextrin, maltodextrin, skim milk, beta-cyclodextrin, gum Arabic, and sodium caseinate in purified water at 22–25°C for 12 h and then dissolving them by using a magnetic stirrer hotplates for 30 min. The mixture consisted of wall material solution, ethanolic extract, and essential oil was homogenized using IKA T18 digital Ultra-Turrax homogenizer (Staufen, Germany) for 5 min at 4000 rpm.

### 3.5. Spray-Drying Conditions

The prepared liquid feeds were spray-dried using a Buchi B-291 Mini Spray-Dryer (BÜCHI Labortechnik AG, Flawil, Switzerland) under the following experimental conditions: according to previous pilot studies inlet temperature was 160 °C, outlet temperature was 80–90 °C, spray flow feed rate—30 mL/min, air pressure—6 bar, aspirator—100%. All of the spray-dried powders were collected in glass vials and stored in a refrigerator at +4–7 °C, where they were protected from light and gas permeation in order to minimize possible changes in the material, such as an agglomeration, caused by oxidation and water absorption.

### 3.6. Analysis of the Spray-Dried Powder

The powders obtained by spray-drying were analyzed for their moisture content, wettability, solubility, bulk and tapped volumes, product yield, encapsulation efficiency, and morphology.

### 3.7. Moisture Content

The moisture content of the spray-dried powder was determined by estimating the powder’s weight loss after oven drying at 105 °C until a constant weight was obtained [26].

### 3.8. Wettability

Wettability of the spray-dried powder was determined according to Antonio et al. [41]. A small amount (1 g) of spray-dried powder was poured into a beaker with 100 mL of purified water and left at room temperature without mixing. The time required for the powder particles to precipitate, to sink, or to become immersed and disappear from the surface of the water was used in a comparison of the extent of wettability between samples.

### 3.9. Solubility

The solubility of spray-dried powder was obtained according to Antonio et al. [35] with some modifications. A small amount (1 g) of the powder was mixed with 25 mL of purified water for 5 min using a mechanical stirrer. The solution was transferred to a tube and centrifuged at 3000× *g* for 10 min at 25 °C. After centrifugation, the supernatant (20 mL) was transferred to the pre-weighed Petri dishes and dried overnight in an oven at 105 °C. The solubility (%) of the spray-dried powder was calculated as the percentage of dried supernatant in relation to the amount of microcapsules by the equations:

(1) $$\mathrm{Solubility}\;\left(\%\right)=\frac{\text{Residue after drying}}{\text{Theoretical residue after drying}}\times 100$$

(2) $$\text{Theoretical residue}=\frac{W_{\text{supernatant to be dried}}-W_{\mathrm{microcapsules}}}{W_{\mathrm{microcapsules}}-W_{\text{purified water}}}$$

where W represents weight.

### 3.10. Bulk and Tapped Volumes

Bulk and tapped volumes (V_0_ and V_tapped_) of spray-dried powder were measured using the density tester (SVM 102 Erweka, Germany) by the method described in Pharmacopeia (Ph. Eur., USP). Obtained values were then used to calculate Carr index (CI) and Hausner ratio (HR):

(3) $$\mathrm{Carr}\;\mathrm{index}\;\left(\mathrm{CI}\right)=\frac{100\times \left(V_{0}-V_{\mathrm{tapped}}\right)}{V_{0}}$$

(4) $$\mathrm{Hausner}\;\mathrm{ratio}\;\left(\mathrm{HR}\right)=\frac{V_{0}}{V_{\mathrm{tapped}}}$$

### 3.11. Spray-Dried Product Yield and Microencapsulation Efficiency

The yield of collected spray-dried product was expressed as the ratio (%) of the mass of powder obtained in the spray-dryer output and the solid content of the initial liquid feed solution using the following definition [42]:

(5) $$\mathrm{Yield}\;\left(\%\right)=\frac{\text{Mass of the powder obtained at the spray-dryer}}{\text{Solid content of the initial feed solution}}\times 100$$

The encapsulation efficiency was determined as the ratio of the concentration of an encapsulated active substance (practical load) to its initial concentration at the beginning of the encapsulation process (theoretical load). Encapsulation efficiency was calculated by these equations [43]:

(6) $$\mathrm{Encapsulation}\;\mathrm{efficiency}\;\left(\%\right)=\frac{\text{Practical load}}{\text{Theoretical load}}\times 100$$

(7) $$\mathrm{Theoretical}\;\mathrm{load}\;\left(\%\right)=\frac{W_{\mathrm{Total}\;\mathrm{drug}\;\left(\text{active compound}\right)}}{W_{\text{dry residue of total drug}}+W_{\mathrm{total}\;\mathrm{excipients}\;\left(\text{wall material}\right)}}$$

(8) $$\mathrm{Practical}\;\mathrm{load}\;\left(\%\right)=\frac{W_{\mathrm{drug}\;\left(\text{active compound}\right)\;\mathrm{in}\;\mathrm{microparticles}}}{W_{\mathrm{microparticles}}}$$

where W is weight.

### 3.12. Powder Preparation for HPLC Analysis

For determination of RA, CA, AP, EK, and DK, the spray-dried powders (100 mg) were dispersed in 10 mL of a mixture composed of methanol and ethanol (*v*/*v*) at ratio 1:1. The solution was sonificated for 10 min and filtered through a 0.22 μm membrane filter for the analysis described below.

### 3.13. HPLC Conditions for Determination of Rosmarinic Acid, Chlorogenic Acid, and Apigenin

HPLC analysis have been carried out according to Pudziuvelyte et al. [34] method. For analysis a Waters 2695 chromatography system (Waters, Milford, CT, USA), equipped with a Waters 996 PDA detector was used. Data was collected and analyzed using the Empower-2 chromatographic manager system (Waters Corporation, Milford, MA, USA). For determination of polyphenols, an ACE 5C_18_ 250 × 4.6 mm (Advanced Chromatography Technologies, Aberdeen, Scotland, UK) column was used. The mobile phase consisted of solvent A (phosphoric acid/acetonitrile/water) (1:19:80 *v*/*v*/*v*) and solvent B (phosphoric acid/methanol/acetonitrile) (1:40:59 *v*/*v*/*v*). The linear gradient elution profile was as follows: 100% A—at 0 min, 55% A/45% B—at 20 min, 100% B—from 25 to 26 min, 100% A—from 30 to 31 min. The flow rate was 1.2 mL/min and the injection volume was 10 μL. Absorption was measured at 330 nm. Quantification of phenolic compounds was performed using reference standards of apigenin, rosmarinic acid, and chlorogenic acid. The linear calibration curves were constructed (apigenin R^2^ = 0.999979, rosmarinic acid R^2^ = 0.999551, chlorogenic acid R^2^ = 0.999914), the peak areas were used for quantification. The contents were expressed as μg/g dry weight.

### 3.14. GC-FID Conditions for Determination of Elsholtzia Ketone and Dehydroelsholtzia Ketone

GC-FID analyses have been carried out using Shimadzu GC-2010 gas chromatograph (Shimadzu, Tokyo, Japan) equipped with a Shimadzu autoinjector AOC-20is (Shimadzu, Tokyo, Japan) according to Pudziuvelyte et al. [34] method. The operational conditions were as follows: temperature increased from 70 °C (3 min) to 180 °C (5 min) at 5 °C/min, then increased to 250 °C (3 min) at 10 °C/min and to 315 °C (10 min) at 10 °C/min. A column RXI-5MS (30 m × 0.25 mm i.d. × 0.25 μm film thickness) was used. Split injector temperature: 260 °C. Split ratio: 1:20. Inlet pressure: 104.0 kPa. Carrier gas: helium (purity > 99%), delivered at constant linear velocity 30.1 cm/s. FID (320 °C) gases: helium (flow 40.0 mL/min); air (flow 400.0 mL/min); helium (as make up, flow 30.0 mL/min).

Quantification was carried out by the external standard method. Elsholtzia ketone (C_10_H_14_O_2_, Mw 162 g/mol) and dehydroelsholtzia ketone (C_10_H_12_O_2_, Mw 164 g/mol) contents were calculated using the equation of linear calibration of eugenol (C_10_H_12_O_2_, Mw 164 g/mol). The linear calibration curve was constructed as an area vs. concentration (eugenol R^2^ = 0.9999).

### 3.15. Total Phenolic Content (TPC) and Surface Phenolic Content (SPC) Determination

The total phenolic and surface phenolic contents were obtained following the methods of Tolun, Altintas, and Artik [44] and Saenz, Tapia, Chavez, and Robert [45] with some modifications. The method based on the color change when Folin–Ciocalteau reagent reduced by sodium carbonate and phenolic compounds. A 100 mg of spray-dried powders were weighted and dispersed in 1 mL ethanol/acetic acid/water solution (20:8:42, *v*/*v*). The mixture was stirred using a magnetic stirrer for 1 min and then an ultrasonicator for 20 min at 25 °C. After that the mixture was filtered through a micro filter (0.45 µm). A 100 µL of sample and 2.5 mL of Folin–Ciocalteau reagent were mixed in a tube and leave in the dark place for 5 min. A 2 mL of sodium carbonate solution was poured into the tube than mixed and leave in a dark place for 1 h at 25 °C. TPC was expressed as mg of equivalent gallic acid per gram of microcapsules. The absorbance was measured at 760 nm using a UV/VIS 1800 Shimadzu spectrophotometer (Shimadzu, Japan).

For the determination of SPC of the spray-dried microcapsules, 100 mg of microcapsules was treated with 10 mL of ethanol/methanol solution (1:1, *v*/*v*) and then filtered through a micro filter (0.45 µm). The SPC was determined according to the same method describe previously for TPC determination. The SPC percentage and TPC EE (%) were calculated according to Equations (9) and (10), respectively.

(9) $$\mathrm{SPC}\;\left(\%\right)=\frac{\text{surface phenolic compounds}}{\text{total phenolic compounds}}\times 100$$

(10) TPC EE (%) = 100 − SPC (%)

### 3.16. Scanning Electron Microscopy

The morphological characteristics of the microcapsules produced with different wall materials at the temperature of 160°C were evaluated by scanning electron microscopy (SEM). The small amounts of spray-dried powders were placed on the double-sided tape surface fixed to stubs. Photomicrographs with magnifications of 500×, 1000×, and 6000× were recorded at 5 kV using Hitachi TM 3000 (Tokyo, Japan) scanning electron microscope.

### 3.17. Statistical Analysis

Statistical analysis was performed by one-way analysis of variance (ANOVA) followed by Tukey’s multiple comparison tests using the software SPSS Statistics 20.0 (IBM Corporation, NY, USA). A value of *p* < 0.05 was taken as the level of significance.

### 3.18. Experimental Design

The experimental settings for experimental design for encapsulating agents were performed by statistical mixture experimental design. The experimental design of four components system is conducted by using Design Expert (version 9.0.4.01, Stat-Easy Inc., Minneapolis, USA). A set of candidate points in the design space is selected using D-optimal criterion. In D-optimal criterion, there are restrictions on the component proportions X_1_ that take the form of lower L_1_ and upper U_1_ constraints, to keep the experimenter from exploring the entire simplex region. The mixture of the components was composed of sodium caseinate, skim milk, beta-cyclodextrin, and resistant-maltodextrin. The constraints of the component proportions were from 0.5% to 12% (Table 1). These lower and upper limits of X_j_ are chosen to describe the behavior of the formulations, which have compositions close to that of the best experiment obtained from preliminary work.

The four wall material components and their levels, respective of experimental design in terms of pseudo-components with 20 combinations, including four replications at the central points, are shown in Table 5.

Factors with a significance higher than or equal to 5% (*p* < 0.05) were considered. Response variables of this experimental design were as follows: EE% (encapsulation efficiency) of RA (rosmarinic acid), CA (chlorogenic acid), AP (apigenin), EK (elsholtzia ketone), DK (dehydroelsholtzia ketone), and TPC (total phenolic content). The concentration of wall material compounds in liquid feeds was 20%, inlet temperature was 160 °C and spray flow feed rate 30 mL/min. These parameters were used as a constant in this experimental design. For each response variable the regression models were evaluated.

## 4. Conclusions

In this study, an ethanolic *E. ciliata* herb extract and essential oil were successfully encapsulated by spray-drying technique using skim milk, maltodextrin, sodium caseinate, gum arabic, resistant-maltodextrin, and beta-cyclodextrin as wall materials. All wall materials enabled the formation of microparticles loaded with *E. ciliata* ethanolic extract and essential oil. The morphology of microparticles, the spray-dried powders characteristics, and active compounds encapsulation efficiency were quite dependent on the wall material used. Using different encapsulating agents, some differences in morphology, physical characteristic, and encapsulation efficiency were found. When wall material solution for spray-drying contained 0.54 g of sodium caseinate, 10 g of skim milk, 8.96 g of resistant-maltodextrin, and 0.5 g of beta-cyclodextrin the EE of RA was 85.27%, CA—85.36%, AP—90.51%, EK—67.38%, DK—35.12%, and TPC—62.78%. The use of resistant-maltodextrin as the main encapsulating material, in supplementation with sodium caseinate, skim milk, and beta-cyclodextrin, increased the encapsulation efficiency, solubility, and wettability more than only using one encapsulating agent for spray-drying solution. Microparticles according to SEM micrographs were round-shaped with a smooth surface when using four encapsulating agents for spray-drying solution. The results showed that wall material solution containing these encapsulants is better at encapsulating phenolic compounds than ketones. To achieve higher EE of EK and DK it is necessary to optimize the conditions of the spray-drying process.

## Figures and Tables

**Table 1:** Parameters studied in physical optimization of liquid feeds mixture of wall material for spray-drying.

Code	Wall Material	Low Level (g)	High Level (g)
A	Sodium caseinate	0.5	1
B	Skim milk	6	10
C	Resistant maltodextrin	8	12
D	Beta-cyclodextrin	0.5	1

**Table 2:** Regression coefficients and final equations of the experimental design.

Response (%)	Final Equation	*p* Value	R^2^	R^2^_Adjusted_
RA EE	= −10.00 A + 91.41 B + 64.50 C − 31.00 D	0.002	0.91	0.81
CA EE	= +10.41 A + 93.00 B + 55.29 C − 35.88 D	0.003	0.86	0.74
AP EE	= −5.89 A + 96.38 B + 67.51 C + 34.64 D	0.001	0.92	0.86
EK EE	= +17.89 A + 81.19 B + 50.49 C − 6.43 D	0.001	0.93	0.85
DK EE	= +52.44 A + 31.74 B + 42.73 C + 94.35 D	0.015	0.87	0.74
TPC EE	= −55.73 A + 22.37 B − 65.52 C + 24.38 D	0.002	0.97	0.9

A—sodium caseinate, B—skim milk, C—resistant-maltodextrin, D—beta-cyclodextrin.

**Table 3:** Values of yield (%), moisture content (%), solubility (%), wettability (s), Carr index (%), and Hausner ratio of spray-dried powders using D-optimal mixture design.

Run	ID	Yield (%)	Moisture Content (%)	Solubility (%)	Wettability (s)	Carr Index (%)	Hausner Ratio
1	01M	66.97 ± 0.98	5.46 ± 0.21	99.8 ± 0.03	86 ± 0.03	25.45 ± 0.04	1.341 ± 0.04
2	02M	59.17 ± 0.23	5.01 ± 0.09	97.5 ± 0.05	77 ± 0.05	28.36 ± 0.06	1.396 ± 0.06
3	03M	61.04 ± 0.26	5.27 ± 0.16	99.8 ± 0.02	80 ± 0.06	30.91 ± 0.08	1.447 ± 0.08
4	04M	44.19 ± 0.33	5.01 ± 0.11	97.5 ± 0.04	73 ± 0.05	34.29 ± 0.06	1.522 ± 0.06
5	05M	56.86 ± 0.15	5.41 ± 0.07	85 ± 0.06	72 ± 0.04	24.44 ± 0.07	1.324 ± 0.07
6	06M	54.32 ± 0.43	4.88 ± 0.05	95 ± 0.08	90 ± 0.08	26.19 ± 0.03	1.355 ± 0.03
7	07M	66.25 ± 0.11	4.09 ± 0.04	90 ± 0.07	70 ± 0.09	26.09 ± 0.05	1.353 ± 0.05
8	08M	58.76 ± 0.09	4.58 ± 0.07	77.5 ± 0.09	72 ± 0.03	31.82 ± 0.07	1.467 ± 0.07
9	09M	47.09 ± 0.12	4.95 ± 0.06	99.25 ± 0.02	85 ± 0.04	37.14 ± 0.03	1.591 ± 0.03
10	10M	47.81 ± 0.36	3.72 ± 0.08	92.5 ± 0.04	105 ± 0.09	35.48 ± 0.02	1.55 ± 0.02
11	11M	56.04 ± 0.22	4.68 ± 0.04	95 ± 0.06	93 ± 0.07	28.57 ± 0.03	1.4 ± 0.03
12	12M	46.93 ± 0.17	4.54 ± 0.03	99.8 ± 0.01	66 ± 0.12	25.71 ± 0.06	1.346 ± 0.06
13	13M	46.92 ± 0.15	4.53 ± 0.05	99.7 ± 0.03	66 ± 0.14	25.70 ± 0.04	1.347 ± 0.09
14	14M	45.22 ± 0.17	4.55 ± 0.23	97.5 ± 0.09	62 ± 0.35	25.23 ± 0.06	1.342 ± 0.06
15	15M	47.82 ± 0.23	3.71 ± 0.05	92.5 ± 0.06	105 ± 0.32	35.47 ± 0.07	1.55 ± 0.06
16	16M	44.20 ± 0.13	5.00 ± 0.07	97.5 ± 0.05	73 ± 0.12	34.28 ± 0.09	1.522 ± 0.07
17	17M	46.92 ± 0.41	5.92 ± 0.08	82.5 ± 0.03	76 ± 0.06	27.45 ± 0.04	1.383 ± 0.04
18	18M	59.45 ± 0.09	4.77 ± 0.06	87.5 ± 0.05	97 ± 0.05	28.89 ± 0.06	1.406 ± 0.06
19	19M	59.7 ± 0.08	4.73 ± 0.04	88.75 ± 0.06	83 ± 0.04	31.82 ± 0.07	1.468 ± 0.07
20	20M	52.27 ± 0.24	5.32 ± 0.08	88.75 ± 0.08	68 ± 0.11	28.21 ± 0.02	1.393 ± 0.02

**Table 4:** Numerical optimization of encapsulants amounts using the desirability function.

**Independent Variables**			
	**Amount Limits (g)**	**Predicted Optimized Amount (g)**	
Sodium caseinate (A)	0.5–1	0.54	
Skim milk (B)	6–10	10	
Resistant-maltodextrin (C)	8–12	8.96	
Beta-cyclodextrin (D)	0.5–1	0.5	
**Response Variables**			
**Encapsulation Efficiency**	**Criteria**	**Predicted Mean Value (%)**	**Obtained Mean Value (%)**
Rosmarinic acid (RA)	Maximize	85.37	85.27
Chlorogenic acid (CA)	Maximize	85.06	85.36
Apigenin (AP)	Maximize	89.96	90.51
Elsholtzia ketone (EK)	Maximize	66.44	67.38
Dehydroelsholtzia ketone (DK)	Maximize	34.02	35.12
Total phenolics content (TPC)	Maximize	62.05	62.78

Desirability 0.905.

**Table 5:** The composition of liquid feeds mixture of wall materials (g) for spray-drying.

Run	ID	Sodium Caseinate	Skim Milk	Resistant Maltodextrin	Beta-Cyclodextrin
1	01M	0.50	7.00	12.00	0.50
2	02M	0.50	6.50	12.00	1.00
3	03M	1.00	6.50	12.00	0.50
4	04M	1.00	8.13	10.13	0.75
5	05M	1.00	6.00	12.00	1.00
6	06M	0.88	7.13	11.13	0.87
7	07M	0.63	9.13	9.38	0.88
8	08M	0.50	8.50	10.50	0.50
9	09M	0.75	10.00	8.50	0.75
10	10M	1.00	10.00	8.00	1.00
11	11M	0.50	10.00	8.50	1.00
12	12M	1.00	10.00	8.50	0.50
13	13M	1.00	10.00	8.50	0.50
14	14M	0.63	7.63	11.13	0.63
15	15M	1.00	10.00	8.00	1.00
16	16M	1.00	8.13	10.13	0.75
17	17M	0.75	8.13	10.13	1.00
18	18M	1.00	6.50	12.00	0.50
19	19M	0.50	6.50	12.00	1.00
20	20M	0.50	10.00	9.00	0.50
